# Microsatellite instability: a review of what the oncologist should know

**DOI:** 10.1186/s12935-019-1091-8

**Published:** 2020-01-13

**Authors:** Kai Li, Haiqing Luo, Lianfang Huang, Hui Luo, Xiao Zhu

**Affiliations:** 1 Guangdong Key Laboratory for Research and Development of Natural Drugs, Guangdong Medical University, Zhanjiang, 524023 China; 2 The Marine Biomedical Research Institute, Southern Marine Science and Engineering Guangdong Laboratory Zhanjiang, Guangdong Medical University, Zhanjiang, 524023 China; 3 Cancer Center, The Affiliated Hospital, Guangdong Medical University, Zhanjiang, 524023 China

**Keywords:** MSI, Cancer, MSI-H/dMMR, Microsatellite DNA

## Abstract

The patients with high microsatellite instability (MSI-H)/mismatch repair deficient (dMMR) tumors recently have been reported that can benefit from immunotherapy, and MSI can be used as a genetic instability of a tumor detection index. However, many studies have shown that there are many heterogeneous phenomena in patients with MSI tumors in terms of immunotherapy, prognosis and chemotherapy sensitivity. Here we mainly review the research results of MSI detection methods, the mechanisms of MSI occurrence and its relationship with related tumors, aiming to make a brief analysis of the current research status of MSI and provide comparable reference and guidance value for further research in this field.

## Background

Microsatellite (MS), also called Short Tandem Repeats (STRs) or Simple Sequence Repeat (SSRs), consists of repeated sequences of 1–6 nucleotides [1]. The distribution characteristics are different from 15 to 65 nucleotides tandem repeats of small satellite DNA, which is mainly located near the ends of chromosomes. MS are widely distributed and mostly is located near the coding region and may be located others region like intron or non-coding region. Each MS specific site is composed of two parts: the central core and the peripheral flanks, and the specificity of MS is mainly due to the change in the number of core repeating units.

The mechanism of MS generation is generally believed to be DNA slippage in the process of replication, or mismatch of the basic group of slippage strand and complementary strand in the process of DNA replication and repair, resulting in one or more of the repeating units missing or insert. The normal tissue DNA repair system, called mismatch repair (MMR), can correct in the process of DNA replication errors. However, due to the lack of MMR genes in tumor cells or defects in the process of replication repair, the possibility of gene mutation is increased [2]. It can be seen that MSI is an important factor in the occurrence and development of tumors.

In line with the frequency of MSI, it can be distinguished into three types: high microsatellite instability (MSI-H), low microsatellite instability (MSI-L) and microsatellite stability (MSS) [3]. At present, clinical research tends to classify MSS-L and MSS as one kind. According to the different molecular mechanisms of MSI in colorectal cancer, it can be divided into colorectal cancer (CRC) with no obvious family genetic history and Lynch syndrome with non-polyposis with family genetic history. Early findings by researchers showed that most of the MSI cases are sporadic colorectal cancer, which is caused by epigenetic inactivation of gene expression in offspring on account of the methylation of hMLH1 promoter without the gene mutation. Lynch syndrome is an autosomal dominant tumor syndrome caused by mutations in MMR strains, and it can also cause tumors in other parts of the colon and rectum [4].

Because of the limitation of early MSI detection and the ambiguity of early MSI mechanism, only some specific chemotherapy drugs can be used to treat MSI patients, and the results are not ideal. With the recent development of MSI detection technology and immunosuppressant in tumor therapy, researchers found that MSI-H tumors respond well to immunotherapy. FDA approved PD-L1 (programed cell death ligand 1) blockade Keytruda to treat MSI-H/MMR patients. Scholars began to conduct more in-depth research on MSI detection methods, MSI mechanism, and the relationship between MSI and tumor.

## Methods and progress of microsatellite instability detection

With the implementation of the human genome project, scientists began to further study the genes related to human diseases, found the microsatellite instability associated with it, and sought to detect the relevant methods (Table 1).

**Table 1:** Summary of microsatellite instability detection methods

Detection method	Characteristics	Test items	Accuracy	Refs.
NGS	Accurate results were obtained from a small amount of sample	Nearly 100 MS loci	IMPACT™: 92% F1CDx: 94.6%	Hempelmann et al. [6]
Fluorescent multiplex PCR and CE	Only MSI results are obtained MSI analysis system is based on this method	5 MS sites: BAT-26, NR-21, BAT-25, MONO-27 and NR-24	Gold standard, 100%	Arulananda et al. [11]
IHC	Wide application and strong practicability, but only get the MMR results	The MMR protein: hMLH1, hPMS2, hMSH2, hMSH6	89–95%	Cheah et al. [12]
smMIPs	Accurate and no matching of normal materials are required for certain diseases: colorectal cancer, prostate cancer, endometrial cancer	DNA from tumor tissue	95.80%	Waalkes et al. [17]

*NGS* next-generation sequencing, *PCR* polymerase chain reaction, *CE* capillary electrophoresis, *IHC* immunohistochemistry, *smMIPs* single-molecule molecular inversion probes, *MMR* mismatch repair, *MS* microsatellite

### Next-generation sequencing (NGS)

Owing to errors in the function of MMR during DNA replication, MSI can be liable to emerge. Clinically, MSI can be detected by detecting changes in microsatellite sequences, or by detecting whether four MMR proteins are missing to determine whether there are MMR functional defects. However, some studies have found that only one detection method may lead to misjudgment, but using two detection methods at the same time faces the problems of high sample demand and high detection cost [5]. In order to solve these problems, micro samples for MSI detection by NGS can be used for disposable detection to get the acquaintance with MSI and whether MMR—related genes and Tumor mutational burden (TMB) alter [6]. NGS detection is directly targeted to one hundred known genes for genome sequencing, to test microsatellite instability in tumor tissues. In 2017, MSK’s IMPACT products were approved to detect microsatellite instability in cancer tissues. In comparison with traditional methods, the uniformity of check results of IMPACT can reach more than 92% [7]. The next year, FMI’s NGS product F1CDX was approved by the FDA and can also be used for MSI [8].

### Fluorescent multiplex PCR and CE

The method of Polymerase Chain Reaction (PCR) is to compare the microsatellite loci detected in tumor tissues with normal DNA. And the National Cancer Institute recommended two single nucleotide repeat loci BAT-25 and BAT-26 and three multi-nucleotide repeat loci D2S123, D5S346 and D17S250 as microsatellite markers to determine the status of MSI [9]. The instability of one site is called low microsatellite instability (MSI-L), and the instability of two or more sites is called high microsatellite instability (MSI-H). The instability of all five sites is called microsatellite instability (MSS). This method can directly reflect the status of MSI, but only the MSI genotype can be obtained [10]. By now, fluorescent multiplex PCR and capillary electrophoresis (CE) is used to detect MSI status on DNA molecular chains in normal tissues and tumor tissues of the same patient. Fluorescence multiplex PCR and CE is used to detect genes after amplification after fluorescence labeled PCR amplification. Due to the characteristics of high efficiency, high sensitivity and reliable analysis results, this detection method has become the gold standard for MSI detection. At present, based on fluorescence multiplex PCR and CE, researchers design MSI analysis system [11] to detect MSI in human cells. This method can detect 5 quasi monomorphic sites BAT-26, NR-21, BAT-25, MONO-27 and NR-24 at one time.

### Immunohistochemistry (IHC)

Detection of MMR gene deletion can indirectly reflect the status of MSI. IHC, a method, is adopted to detect the expression of MMR protein which consists of hMLH1, hPMS2, hMSH2 and hMSH6 [12]. If the result shows that any of the above MMR protein expression is absent, it means MMR deficient (dMMR). If all four MMR proteins are expressed, it means Proficient Mismatch Repair (pMMR). In general, dMMR is equivalent to MSI-H [13]. IHC is so simple and practical that some people think it can be used to replace PCR [14, 15]. But in some cases, dMMR and MSI-H could not be detected at the same time. For example, dMMR caused by MSH6 mutation could not meet the criteria of MSI-H diagnosis, and MSI-H positive tumor may come from MMR pathway protein which could not be detected by current technology. Therefore, some studies suggest that the application of molecular analysis to IHC and MSI analysis can reduce the incompatibility of results [16].

### Single-molecule molecular inversion probes (smMIPs)

Recently, the Academy of Sciences published a method to detect microsatellite instability by using smMIPs, which are accurate and do not require patients to match normal materials. This method can accurately diagnose pan cancer microsatellite instability by single molecule reverse probe capture and high-throughput sequencing. According to this study, smMIPs can only accurately identify microsatellite instability in colorectal, prostate and endometrial cancers to determine the presence of MSI [17, 18].

### MSI calculation method

MANTIS is to take the average value as MSI score after calculating the allele distribution difference value of each microsatellite site by comparing tumor and normal samples. MANTIS can detect more MS sites and maintain high accuracy. At present, MANTIS is widely used in the MSI detection of pan cancer [19]. Recently, researchers have built a binary classifier with the core of convolutional neural network (CNN). By developing a deep learning algorithm, resnet18, the researchers used tumor cell detection and he stained histological section images to predict MSI status. Compared with the existing tumor detection data set, resnet18 has the advantages of short training time and good classification performance, but it also has the disadvantages of small amount of training data and single representativeness of training samples. It has not been widely popularized at present [20].

## Mechanism of MSI

### Slipped strand mispairing

In addition to point mutation, MS can also be caused slipped strand mispairing (SSM) [21]. SSM is that in the process of DNA replication and synthesis, the allele region of MS repeat sequence between new chain and template chain may be mismatched, resulting in the instantaneous separation of new chain and template chain or the formation of stable single chain structure by several repeat units. Lai et al. [22] found that the MS sliding mutation rate increased exponentially with the number of repeat units. When slip mutation occurs, MS with small number of repeat units expands more frequently, while MS with large number of repeat units contracts more frequently.

### MMR deficient

The mismatch repair (MMR), can repair errors during DNA replication. For example, in the above slip mechanism, when the mutated MS is paired with another chain, the redundant structure that may be formed after the slip chain can be restored to the level before replication if it is cut by nuclease and repaired, and remain in the new chain if it is not repaired. MMR deficient (dMMR) makes the errors produced during DNA replication impossible to repair, which leads to nucleotide mutation and changes in the length of simple repeat MS sequence [2, 23].

### Characteristics of gene mutation in MSI-H patients

Researchers have found that mutations in MSI-H patients had commonalities. For example, the germline mutations of MMR gene, POLE (polymerase E)/POLD1 (human DNA polymerase δ) are more common in patients with MSI-H than in patients with MSS. It is also found that the tumors with polar MSI-H can improve the translation level of oncogenes by shortening 3 ‘UTRs (3′-untranslated regions), which may cause frequent MSI, but may lead to the loss of miRNA (microRNA) mediated regulation [24]. This could be used to explain the results of a 2015 study. According to the research results, a class of noncoding RNA molecules similar to cancer cell pathogens can trigger human immune response and accelerate the development of cancer. It also found that these noncoding RNA are transcribed by a class of satellite DNA, although they do not produce proteins, their regulatory role is closely related to the growth of tumor [25, 26].

### Mutational characteristics in various cancers

An analysis based on TCGA data shows that there are not only the same repeated loci in MSI-H cases, but also some tumor specific loci. For example, its transmembrane/TGFβ, cell stress response/DNA damage and chromosome/M-phase related molecular functions are abundant in the genes of recurrent MSI, and the occurrence of frameshift MSI in TGFBR2 is more common in Colon adenocarcinoma (COAD) and Stomach adenocarcinoma (STAD) than in Uterine corpus endometrial carcinoma (UCEC), indicating that specific tumor environment is conducive to the occurrence of MSI events [24]. Patil et al. [27] found that with five quasi-singlet markers (NR-21, BAT-25, MONO-27, NR-24 and BAT-26), the Promega MSI analytical reagent can accurately identify MSI-H CRC without pairing normal DNA. Hause et al. [28] suggested that MSI mainly concentrates in some tumors of functional areas. For example, MSI occurs mostly in ion-binding genes in gastric adenocarcinoma. And the study showed that tumor suppressor genes ACVR2A and RNF are the most common and effective mutation targets in MSI-H tumors.

### Treatment mechanism of MSI tumors

With the development of immunosuppressive drugs, it is helpful to study the immune response caused by MSI tumor. Scientists have found that some of the mechanisms of action of drugs suitable for MSI-H treatment, such as PD-L1 (programed cell death ligand 1) immunosuppressant, can produce heteroantigens that are easy to be recognized by T cells in dMMR cancer cells, which is beneficial to a variety of MSI-H tumors [54]. At present, there are researches on specific tumor targets of MSI. After analyzing multiple sets of data through CRISPR-Cas9-mediated knockout and RNA interference, Chan et al. that RecQDNA helicase WRN (Werner syndrome, RecQ helicase-like) was an essential factor for the MSI model, but not an important factor for microsatellite stable tumors. Silencing WRN can induce DNA double-strand breakage, activate DNA damage response, induce apoptosis and cell cycle arrest MSI tumors require WRN helicases but do not cause death of their own cells, suggesting that WRN may be a target for lethal synthesis [29].

The above studies indicate that microsatellite mutation is a multi-pathway process, and the continuously updated and developed MSI mechanism will play a more important role in the diagnosis and treatment of future clinical applications.

## Advances in the clinical application of microsatellite instability

### MSI-H/dMMR related diseases

Understanding the classes of diseases (Table 2) associated with MSI is the basis of diagnosis and cure of illnesses with MSI-related technologies.

**Table 2:** Summary of MSI-H/dMMR related diseases

Diseases	MSI characteristics	Prognosis	Treatment options
Lynch syndrome (LS)	EpCAM immunostaining is an important factor, common in patients over 60 years old, most of them arenormal adenocarcinoma, villous adenoma, adenoma over 1 cm and highly dysplastic adenoma	MSI-H/dMMR patients with lynch syndrome have good prognosis	Aspirin/sulinda may play a preventive role in reducing the risk of Lynch syndrome-related cancer, especially in patients with hMSH2 and hMLH1 gene changes
Colorectal cancer (CRC)	MSI-H tumors are infiltrated with dense cytotoxic T cells, generally occur on the right side	Stage I and stage II MSI CRC have good prognosis, stage III MSI CRC have bad prognosis	Stage III to IV CRC patients can use 5-FU as a chemotherapeutic, neither stage I to II CRC patients. And choose anti-PD-1/PDL-1 treatment for CRC patients at different stage
Gastric cancer (GC)	The high expression of CD8 positive T cell molecular marker, PD-L1 gene and IFN γ gene in patients with MSI-H	MSI-H resectable primary gastric cancer have good prognosis	MSI-H GC should avoid adjuvant chemotherapy, take surgical treatment
Breast cancer	BRCA1 mutation can cause MSI. MSI related loci D3S1766 and D2S2739 can identify MSI related breast cancer	MSI-H patients with breast cancer have bad prognosis	Olaparib can strengthen other drugs’ effect such as platinum in combination
Prostate cancer	MSI-frequency < 1%, is closely related to pathogenic embryonic mutants carrying Lynch syndrome-related genes	MSI-H/dMMR patients with prostate cancer have good prognosis	Anti-PD-1/PDL-1 treatment
Cholangiocarcinoma	MSI frequency < 1%, most of them are young patients with atypical tissue morphology	MSI-H/dMMR patients with cholangiocarcinoma have good prognosis	ICI (immune checkpoint inhibitor) combined with radiotherapy
Leukemia	MSI frequency < 1%, most of them are chronic myeloid leukemia	MSI-H/dMMR patients with leukemia have good prognosis	Anti-PD-1/PDL-1 treatment
Bladder cancer	hMSH2mutation can increase the risk of getting bladder cancer, MSI related loci D9S63, D9S156, and D9S283 can be used to screen patients with high micro bladder cancer	MSI-H/dMMR patients with bladder cancer have good prognosis	Anti-PD-1/PDL-1 treatment
Ovarian cancer	An increased number of CD8+, PD-1+, and TILS in MSI Ovarian cancer patients	the MSI-H patients with Clear-cell ovarian carcinoma (CCOCs) are suitable for immunotherapy	Anti-PD-1/PD-L1 drugs
Endometrial Carcinoma (EC)	UCEC patients with MSI has higher immune components, CD3+ and CD8 + TIL	MSI-H EC in the middle and late stage is associated with bad prognosis	Use anti-PD-1/ PD-L1 drugs and chemotherapeutic drugs such as temozolomide and cisplatin.
Pancreatic ductal adenocarcinoma (PDAC)	HMLH1 and hMSH2 are mostly inactivated	MSI-H/dMMR patients with PDAC have good prognosis	Anti-PD-1/PD-L1 drugs
Follicular thyroid cancer (FTC)	Advanced FTC associated with MMR inactivation	MSI-H patients with FTC have a prolonged survival time	Anti-PD-1/PD-L1 drugs
Adrenocortical cancer (ACC)	MSI-H/dMMR patients with ACC have high variation load, ACC is closely related to the deletion mutations of hMSH2	no relevant literature about the effect of MSI on the prognosis of cortical carcinoma	ACC is not effective in immunotherapy of dendritic cells without immune response

*PD-L1* programmed cell death-Ligand 1, *dMMR* mismatch repair deficient, *MSI-H* microsatellite high instability

#### Lynch syndrome

**Fig. 1:**
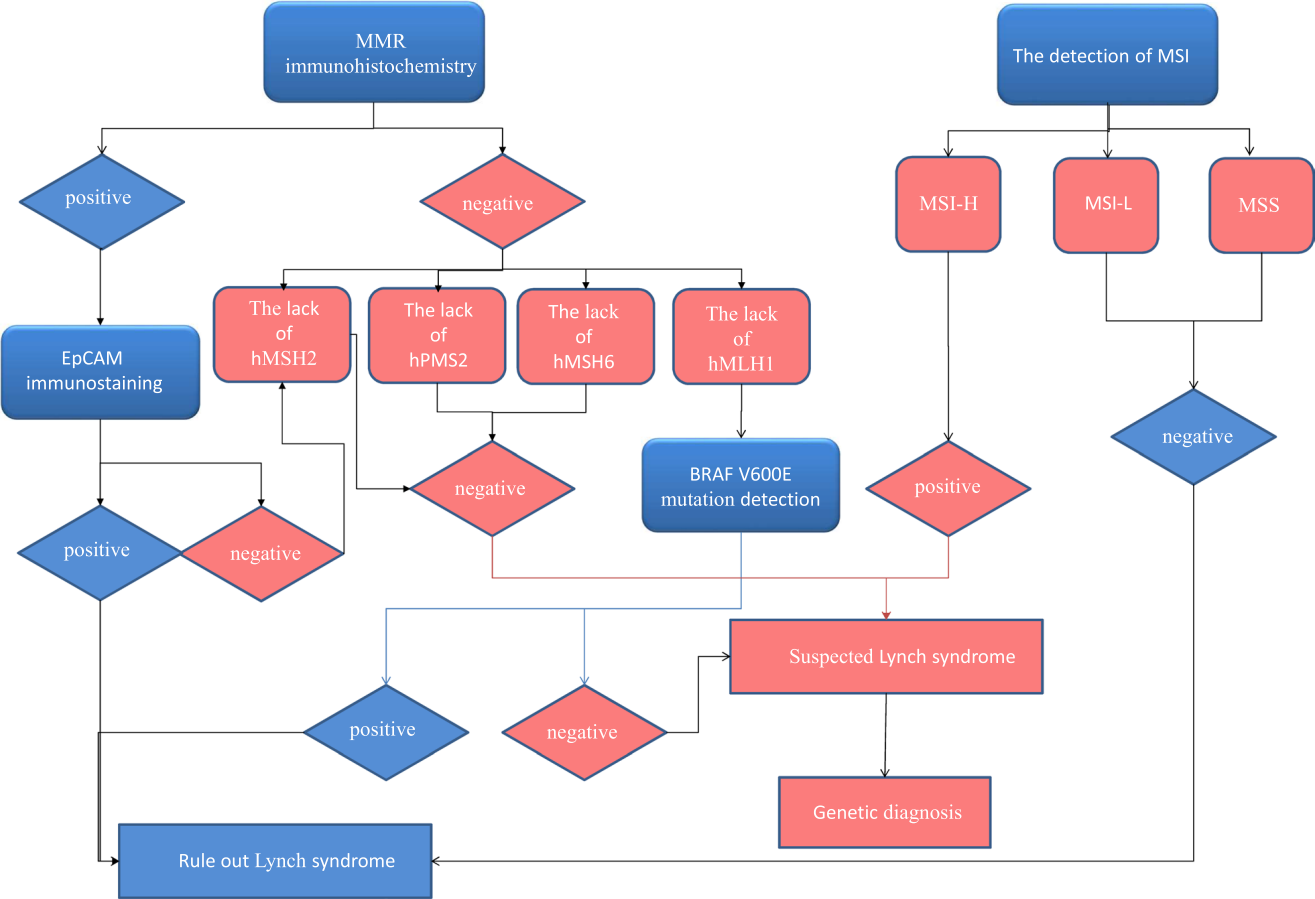
Lynch syndrome screening process. Lynch syndrome screening process. MMR immunohistochemical method is used to detect whether 4 MMR proteins of hMLH1, hPMS2, hMSH2 and hMSH6 are missing, and whether BRAF V600E mutation exists in hMLH1 negative protein and whether EpCAM mutation exists in hMLH1 positive protein, so as to determine whether there is MMR functional defect. MSI detection is to determine the stability of MSI sites by detecting nucleotide marker: two of the unstable loci were MSI-H, the instability of one of the loci was MSI-L, MSS was defined as the instability of zero loci. *MMR* mismatch repair, *MSI-H* microsatellite high instability, *MSI-L* microsatellite low instability, *MSS* microsatellite stability

Latham et al. [30] reported that dMMR is common in patients with Lynch syndrome (LS), so patients with MSI-H or dMMR tumors can predict Lynch syndrome through MSI related tests. NCCN guidelines also recommends gene testing for Lynch syndrome, including MMR genes (hMLH1, hMSH2, hMSH6, hPMS2) and EpCAM genes. MMR IHC screening and MSI detection screening are two ways to screen for patients with Lynch syndrome (Fig. 1). The IHC test results show that the negative hMLH1 cannot directly indicate that there is no mutation in hMLH1, and the hMLH1 promoter needs to be tested to determine whether there is methylation or BRAF (v-RAF murine sarcoma viral oncogene homologB1) mutation, so as to exclude Lynch syndrome [31]. However, EpCAM (Epithelial cell adhesion molecule) gene [21] is still needed to be detected in suspected hMSH2 patients, because LS caused by hypermethylation of hMSH2 which caused by EpCAM body mutation will also lead to the loss of MMR protein expression in IHC detection, but hMSH2 mutation analysis is negative. Only when EpCAM immunostaining is negative, EpCAM abnormality indicates hMSH2 mutation [32]. MSI was detected by nucleotide markers: two of the unstable sites were MSI-H, the instability of one of the locus was MSI-L, locus-free instability was MSS [33]. But some diseases need to be identified by multiple detection methods at the same time. For example, Cosgrove et al. [34] found that the Lynch syndrome could not be recognized by IHC alone or MSI, and the evaluation of endometrial cancer by MSI, hMLH1 methylation and IHC combined application was needed. A research of Dabir et al. [35] stated MSI-H was more common in patients over 60 years old with LS, and most of them were found in normal adenocarcinoma, villous adenoma, adenoma over 1 cm and highly dysplastic adenoma.

#### Colorectal cancer

MSI is closely related to colorectal cancer (CRC), and some studies have found MS loci related to CRC. Saeterdal et al. [36] clarified that between high-level microsatellite instability colorectal cancer patients, compared with MSS tumors, tumors are infiltrated with dense cytotoxic T cells. The study of Nouri Nojadeh et al. [37] confirmed that NR-21, BAT-26 and BAT-25 markers play an important role in judging MSI status in CRC. Promega MSI analytical reagent [38], which has five quasi-singlet markers (NR-21, BAT-25, MONO-27, NR-24 and BAT-26), can accurately identify MSI-H CRC without matching normal DNA at present. BRAF mutation affects the MMR function of early diseases, and has an important effect on CRC. Fujiyoshi et al. [39] found that the prognosis of MSI-H colorectal cancer was good, which was the important reason of BRAF mutation in early diseases, while the prognosis of MSS and MSI-L CRC was poor. However, a study of Goldstein et al. [40] showed that BRAF mutation is associated with MSI-H in metastatic CRC patients with advanced BRAF mutation,but BRAF mutation is a bad factor. According to the article of Carr et al. [41], lifestyle can affect the molecular pathological types of colorectal cancer and statistics show that moderate and high alcohol consumption is associated with an increased risk of MSI-H colon cancer. MSI can be used as a significant molecular marker for prognosis and adjuvant therapy of CRC [42]. MSI has different effects on the lymph nodes and distant metastasis of CRC in different periods, and on the prognosis of patients. Other studies suggested that patients with stage I and stage II MSI colorectal cancer have good prognosis, high 5-year survival rate, low recurrence rate and deterioration rate, but patients with stage III MSI colorectal cancer have the opposite results [43]. Arakawa et al. [44] suggested that most of the tumors in MSI-H colorectal cancer patients occur on the right side.

#### Gastric cancer

Choi et al. [45] suggest that microsatellite instability is also found in gastric cancer. Using hMLH1 and hMSH2 in IHC and MSI analysis system, patients with MSI related gastric cancer can be detected [46]. Small intestinal adenocarcinoma is the most common type of gastric cancer in Lynch individuals. Imamura et al. [47] found that MSI esophagogastric junction adenocarcinoma (EGJ) which is a special gastric cancer is closely related to genetic instability. And the researchers suggested that tumors in Siewert type I are not related to MSI, while tumors in Siewert type II and III are related to MSI. Smyth et al. [48] illuminated that the survival time of patients with MSI-H gastric cancer who can be operated on is not better than that of patients with MSI-L or MSS. Because the American Society of clinical oncology gastrointestinal cancer symposium found that MSI can be used as a good prognostic indicator for resectable primary gastric cancer, future clinical trials need to consider whether to use immunosuppressive checkpoint inhibitors (ICI) to treat gastric cancer with high microsatellite instability due to MSI as a stratification factor [49]. For the mechanism of ICI treatment, one study found that it may be because of the high expression of CD8 positive T cell molecular marker, PD-L1 gene and IFN γ (interferon-γ) gene in patients with MSI-H. Marrelli et al. [50] stated that the tumor of MSI-H patients with gastric cancer is common in women, and most of them occur in non-cardia area.

#### Breast cancer

Abida et al. [51] found that the prognosis of MSI-H patients with breast cancer is poor, which is different from the relationship between MSI and prognosis of breast cancer. IHC detection of hMSH2, hMLH1 and MSI related loci D3S1766 and D2S2739 can identify MSI related breast cancer [52]. At present, the clearest influencing factor is BRCA1. A study of Zhu et al. has shown that BRCA1 expression function can affect the silencing mechanism of satellite DNA in chromatin, which will be damaged by BRCA1 mutation, resulting in failure of normal cell replication process. At the same time, the mutation of BRCA1, which can make the repair function of DNA loss, will cause microsatellite instability and abnormal cells [53].

#### Prostate cancer

An analysis of prostate cancer has shown that MSI-H/dMMR phenotypes can be detected in some prostate cancer patients, including pathogenic embryonic mutants carrying Lynch syndrome-related genes. Using IHC to detect MMR related proteins and MSI analysis and NGS can improve the accuracy of MSI related prostate cancer recognition [6, 54]. Although this phenomenon is not common, it has therapeutic significance. And the study of Abida et al. showed that MSI-H/dMMR patients with bladder cancer have good prognosis for the anti-PD-1/PD-L1 treatment [55].

#### Cholangiocarcinoma

Goeppert et al. [56] have found that MSI-H can be detected in a small number of cholangiocarcinoma patients who are not related to liver trematode. Patients with MSI related cholangiocarcinoma can be detected by analyzing MSI related loci BAT25, BAT26, and CAT25 [56]. And they found that most of the patients with MSI-H were young patients with atypical tissue morphology. And they also suggested that MSI-H/dMMR patients with cholangiocarcinoma have good prognosis for the anti-PD-1/PD-L1 treatment.

#### Leukemia

Walker et al. [57] discovered that MSI can’t be detected in patients with acute myeloid leukemia. However, Patel et al. [58] clarified that MSI was detected in patients with chronic myeloid leukemia, while there was no MSI in normal people, so it was speculated that there was a certain relationship between chronic myeloid leukemia and microsatellite instability. They found that analysis of MSI related loci D17S261 and D3S643 is helpful to identify MSI related chronic leukemia. And his study suggested that MSI-H/dMMR patients with chronic myeloid leukemia have good prognosis for the anti-PD-1/PD-L1 treatment.

#### Bladder cancer

IHC detection of hMLH1, hMSH2 and hMSH6 and MSI analysis can detect MSI associated bladder cancer [59]. A study of Skeldon et al. [60] has indicated that patients with MSI-H Lynch syndrome have a rising risk of bladder cancer because of hMSH2 mutations. However, one study of Giedl et al. [59] demonstrated that a lot of young patients have a low risk of bladder cancer when they get MSI-H Lynch syndrome. Though researcher had found only a few number of bladder cancer patients were diagnosed with MSI-H, these patients can benefit from anti-PD-1/PD-L1 treatment. Zekri et al. [61] suggested that the MSI related loci D16S476, D9S171 of MSI-H patients with bladder cancer are consistent with those in urine, and the MSI of urine sediment can be considered as a clinical assistant diagnosis. Wadhwa et al. [62] stated that MSI related loci D9S63, D9S156, and D9S283 can be used to screen patients with high micro bladder cancer.

#### Ovarian cancer

A research of by Howitt et al. [63] made known that an increased number of CD8+, PD-1+, and TILS in MSI patients. Compared with MSS, the patients with Clear-cell ovarian carcinoma (CCOCs) are likely to benefit from immunotherapy. MSI analysis and MMR related protein detection of hMSH2 and hMSH6 can be used to identify MSI ovarian cancer [64].

#### Endometrial carcinoma

MSI analysis and IHC MMR related protein detection can be used to identify MSI endometrial carcinoma. Compared with MSS, UCEC patients with MSI have higher immune components, CD3+ and CD8+ TIL. A study of Howitt et al. [65] has demonstrated that the immune blocking effect of the tumor itself is great relative to generate immunosuppressive microenvironment. Llosa et al. found that this immune function is caused by the upregulation of several checkpoint ligands, among which PD-1/PD-L1 is an important ligand [66]. The prognosis of early Endometrial Carcinoma (EC) is not correlated with MSI [67], but the results of EC in the middle and late stage were opposite. Bilbao et al. showed that the intermediate and advanced endometrial cancer of MSI was associated with poor prognosis index [34, 68].

#### Pancreatic ductal adenocarcinoma

Wilentz et al. showed that MSI-H can be detected in a small number of Pancreatic ductal adenocarcinoma (PDAC) patients [69]. MSI can be detected in Medullary carcinomas of the pancreas and Acinar cell carcinomas of the pancreas. And hMLH1 and hMSH2 inactivation have been detected in many pancreatic cancer patients [70]. Yamamoto et al. found that PDAC patients with dMMR/MSI have a significantly good prognosis [71].

#### Thyroid cancers

Using NGS and IHC, MSI analysis can detect patients with MSI related thyroid cancer. Genutis et al. reported that MSI-H can be detected in patients with thyroid cancer, especially in patients with follicular thyroid cancer (FTC) [72]. It is also considered to be associated with delayed MMR inactivation in advanced thyroid cancer. And MSI-H patients with FTC have a prolonged survival time.

#### Adrenocortical carcinoma

Some studies have found that adrenocortical cancer (ACC) is related to MSI. McCabe et al. showed that the occurrence of MSI in adrenocortical carcinoma is related to the deletion mutations of MSH2 [73]. A study of Bonneville et al. suggested that MSI-H/dMMR patients with adrenocortical cancer have high variation load [74]. There is no relevant literature about the effect of MSI on the prognosis of cortical carcinoma.

### Preventive measures for MSI-H/dMMR related diseases

As research into MSI-H/dMM related diseases developing, scientists are attempting to search for ways to prevent them. According to the NCCN guidelines, MSI or MMR testing should be considered for all types of colorectal cancer. And early detection of MSI or MMR and prophylactic polypectomy can reduce CRC mortality [75]. Ruschoff et al. [76] suggested that aspirin/sulinda may play a preventive role in reducing the risk of Lynch syndrome-related cancer by reducing microsatellite instability in colorectal cancer cells, especially in patients with hMSH2 and hMLH1 gene changes. Data research showed that HLA-A0201-restricted cytotoxic T cell epitope (FSP11) is expected to become an integral part of MSI-H tumor vaccine in the future, because MSI-H -related transfer peptide (FSP) can induce anti MSI-H tumor response [77].

### The treatment of MSI-H/dMMR related diseases

For patients with MSI-H/dMMR related diseases, scientists are seeking some precise treatments. As early as in the 2011 NCCN guidelines, MSI detection was required before the use of 5-FU as a chemotherapeutic agent in patients with CRC. Warusavitarne et al. [78] reported that 5-FU is not used as adjuvant chemotherapy for patients with MSI-H and dMMR characteristics for the reason that patients with MSI-H/dMMR have poor results after 5-FU adjuvant chemotherapy. Sargent et al. [79] showed that the effect of adjuvant chemotherapy with fluorouracil monotherapy for stage II CRC patients with MSI-H is not good, so it is not necessary to use fluorouracil monotherapy as adjuvant therapy for stage II CRC patients with MSI-H. However, the study of Benson et al. [80] stated that the MMR status of patients with stage III to IV CRC does not affect the outcome of 5-FU treatment. No matter whether the MMR function is abnormal, 5-FU has a curative effect for the MMR status of patients with stage III to IV CRC [81].

Some studies have shown that due to dMMR cancer cells can produce heterologous antigens that are easily recognized by T cells,the effectiveness of PD-1 inhibitors on solid tumors expressing MSI-H is higher than that of solid tumors expressing MSI-L and MSS [82]. Keytruda is therefore approved by FDA for solid tumors with MSI-H/dMMR characteristics based on the biomarkers contained in the tumors [83]. MSI-H can be used as one of the predictors of the efficacy of immunotherapy at present. Phase III clinical trial IMblaze370 made clear that the PD-L1 inhibitor Atezolizumab alone or combined with the mek1/2 inhibitor Cobimetinib did not work well, because the PD-1/PD-L1 inhibitor requires a sufficient amount of new antigen to be recognized by T cells for its effect. We can also find this in Mark Yarchoan’s article that the high benefit rate of dMMR patients on PD-1/PD-L1 inhibitors may be related to the gradual accumulation of mutations [84]. The drug treatment of colorectal cancer is developing constantly (Table 3). FDA approved Nivolumab for patients over 12 years of age with advanced metastatic colon cancer who developed MSI-H or dMMR after previous treatment with fluoropyrimidine, oxaliplatin or irinotecan [85, 86]. Overman et al. [87] suggested that Nivolumab is effective not only for patients with dMMR/MSI-H metastatic colorectal cancer, but also for those with poor prognosis of BRAF mutation in CRC. It can be inferred that Nivolumab is also effective for dMMR/MSI-H metastatic colorectal cancer caused by BRAF mutation. The FDA approved the combination therapy of Nivolumab and Ipilimumab as the first immunosuppressive combination therapy for metastatic CRC with worsened MSI-H/dMMR after treatment with oxaliplatin, irinotecan and fluoropyrimidine [88]. The results of CheckMate-142 trial showed that low dose ipilimumab combined with nivolumab can reduce the toxic and side effects of patients, and can play a better therapeutic effect on patients with partial metastatic CRC, so it can be used as a new first-line treatment [89, 90]. A study by O’Neil et al. [91] showed that Pembrolizumab is safe and less adverse events for patients with advanced PD-L1 positive CRC. It can be used as a drug of choice for patients with advanced anti-PD-L1 positive CRC. One study of Innocenti et al. [92] found that chemotherapy plus Cetuximab was significantly less effective than chemotherapy plus bevacizumab in the treatment of MSI-H patients. They hypothesized that, there are different reasons for the efficacy of bevacizumab and cetuximab. The reduced efficacy of the EGFR inhibitor (cetuximab) is due to the adverse effect of the over methylation of the MSI-H tumor on the EGFR inhibitor, resulting in the low expression of the EGFR ligand. Because the vascular normalization induced by bevacizumab can enhance the infiltration and activation of Th1 lymphocytes, thereby achieving immune stimulation and enhancing the anti-tumor effect of cellular immunity, bevacizumab is more effective than cetuximab in the treatment of MSI-H tumor patients.

**Table 3:** Summary of drug therapy for colorectal cancer patients with MSI characteristics

Drug	Target	Indications	Remarks	Refs.
Fluorouracil	Nucleic acid	Stage III to IV CRC	Chemotherapy	Kwon et al. [81]
Nivolumab	PD-L1	Patients with advanced metastatic CRC over the age of 12 years with MSI-H/dMMR; BRAF mutation caused by dMMR/MSI-H metastatic CRC	Based on the chemotherapy drug treatment	Overman et al. [87]
Ipilimumab	CTLA4	Combined treatment with Nivolumab for metastatic CRC aggravated by MSI-H/dMMR after oxaliplatin, irinotecan and fluorouracil	Low dose ipilimumab combined with nivolumab can reduce side effects in patients	Overman et al. [89]
Pembrolizumab	PD-L1	Advanced anti-PD-L1 positive CRC patients	Based on the chemotherapy drug treatment	O’Neil et al. [91]
Bevacizumab	VEGF	Patients with MSI-H tumor	Based on the chemotherapy drug treatment	Innocenti et al. [92]

*PD-L1* programmed cell death-Ligand 1, *CTLA4* cytotoxic T-lymphocyte-associated protein 4, *VEGF* vascular endothelial growth factor, *CRC* colorectal cancer, *dMMR* mismatch repair deficient, *MSI-H* microsatellite high instability

There are new developments in the treatment of other MSI-H tumors. Recent research of Kim et al. [93] proposed that PD-1 inhibitors can be used to treat patients with metastatic gastric cancer with positive MSI-H. As for whether adjuvant chemotherapy drugs or not, it has been found that chemotherapy has no significant effect on the prognosis of gastric cancer patients with MSI-H, and the expression of PD-L1 is related to the better survival of patients, but its potential benefits of PD-L1 may be weakened due to chemotherapy, so it is suggested that patients with MSI-H should avoid adjuvant chemotherapy [94]. A data analysis of Pietrantonio et al. in the same year also showed that chemotherapy was not good for the prognosis of MSI patients with gastric cancer, and pointed out that for all patients with local advanced gastric cancer who considered to receive adjuvant chemotherapy, we should detect their MSI, and for MSI patients, we should consider to avoid chemotherapy and only take surgical treatment [20]. Olaparib which can inhibit the repair of DNA damage in cancer cells is related to reduce MSI as well as promote apoptosis, and strengthen other drugs’ effect such as platinum in combination, and then achieve the effect of treatment of breast cancer [95]. Although immunosuppressive agents can be applied to patients with MSI-H tumors, some studies have found that the number of patients with MSI-H tumors who benefit from immunosuppressive agents is less. Recently, however, it has been proposed that ICI combined with radiotherapy can reduce the incidence of adverse events in patients with advanced biliary tumors. ICI combined with radiotherapy is recommended as a treatment plan for patients with advanced biliary tumors [96]. Researchers conducted MSI sensor score analysis on 1033 patients with prostate cancer and found that a total of 32 patients had MSI-H tumor [55]. Among them, more than half of the patients had stable condition or remission after receiving anti-PD-1/PD-L1 treatment. In 2016, Fader published a clinical study on Pembrolizumab for PD-1 blockade in patients with dMMR EC, providing a new therapeutic direction for patients with dMMR EC. ASCO 2017 reported that anti-PD-1/PD-L1 drugs have great potential in EC treatment. The mechanism of chemotherapeutic drug resistance to tumor has been further discovered. Fader et al. [97] found that the drug resistance is mainly related to dMMR. The DNA self-repair function of tumor cells can resist the damage of chemotherapeutic drugs such as temozolomide and cisplatin to the DNA function of tumor cells, so as to achieve the effect of drug resistance. A study of Habra et al. have shown that ACC is not effective in immunotherapy of dendritic cells without immune response [98].

## Relationships between MSI and TMB

MSI is due to MMR deletion or gene replication process deletion or error, leading to changes in the length of MS. TMB refers to tumor mutational burden, representing the number of mutations per million bases. Both MSI and TMB represent the production of new antibodies. Studies have stated clearly that there are many patients with MSI-H who have high TMB levels [99]. And Bonneville et al. found that MSI-H adrenocortical carcinoma and cervical squamous cell carcinoma have obvious high mutation [74]. Tumors with high mutation rates may respond well to checkpoint inhibitors (CPI), so we can consider using CPI to treat MSI-H patients with TMB [100].

## Conclusions and perspectives

A large number of studies have shown that MSI is closely related to tumor. The development of fluorescence multiplex PCR and CE and IHC promote the development of MSI detection, but there are also factors affecting the accuracy, considering whether it is related to the MMR protein that cannot be detected at present [16]. For example, if the dMMR caused by MSH6 mutation cannot meet the criteria of MSI-H diagnosis, scientists need to increase MS loci other than BAT-25 and BAT-26 and three multi nuclear repeat loca D2S123, D5S346 and D17S250 to improve the accuracy of MSI. The latest smMIPs [17] and deep learning from histology in tumor are very promising for clinical application of MSI detection. However, smMIPs can only be used for the detection of chromatic cancer, prostate cancer, and objective cancer and deep learning from history in tumor [20] still needs a large number of clinical experiments to improve the accuracy. Therefore, NGS can be considered [6], despite IHC and PCR are still widely used.

Early diagnosis of MSI is of great significance to the prognosis and treatment of MSI. At present, it is found that there is a certain correlation between MSI related tumor and clinical phenotype, which can be detected early by MSI in high-risk MSI patients. For example, the tumor of MSI-H patients with gastric cancer generally occurs in non-cardia area [50]. Because of tumor heterogeneity, the prognosis of different MSI is different. For example, the prognosis of most MSI-H cancer patients is good, while the prognosis of breast cancer [51] and endometrial cancer [67] patients with MSI-H is bad. Besides, early diagnosis can help to take preventive measures for its diseases, such as Lynch syndrome, and early use of aspirin in patients with hMSH2 and hMLH1 gene changes is of great significance to reduce the risk of cancer related to Lynch syndrome [76]. At the same time, early development of vaccines that can induce anti-tumor response is also of great significance for the prevention of MSI-H tumors. For example, the latest discovery of FSP11, which can be an important component of vaccines [77].

Although some studies have found the specific MS site of MSI tumor, but because MSI is a multi-channel process, it is still complex to study the disease caused by MSI and the treatment mechanism of MSI tumor. It has been found that MSI leads to drug resistance of tumor [97]. More clinical studies on the relationship between MSI related sites and tumor drug resistance are needed to improve the therapeutic effect of chemotherapy. For MSI-H patients with TMB, CPI inhibitors can be considered for treatment of MSI-H tumors [100]. With immunotherapy widely used in tumor treatment, a large number of data show that MSI-H can be used in immunosuppressive therapy, MSI can be used as an effective positive immunotherapy predictor. But the benefit of tumor is different in different periods. For example, nivolumab is suitable for patients with advanced metastatic colorectal cancer [85]. If the newly found synthetic lethal target silencing WRN [29] can be used in clinical treatment, more MSI patients will benefit in the future.

In general, the detection method of MSI, the mechanism of MSI and its relationship with related tumors have made progress. However, it is still necessary to detect MSI in rare tumors, and improve the number of MSI related tumors and the classification of tumors. It is believed that with the more accurate detection technology of MSI and the clearer relationship between the mechanism of MSI and MSI-related tumors, MSI will open up a new field for the diagnosis, prevention and treatment of diseases.

## Data Availability

Not applicable.

## Abbreviations

CE: capillary electrophoresis
CRC: colorectal cancer
CTLA4: cytotoxic T-lymphocyte-associated protein 4
dMMR: mismatch repair deficient
IHC: immunohistochemistry
MMR: mismatch repair
MS: microsatellite
MSI-H: microsatellite high instability
NGS: next-generation sequencing
PCR: polymerase Chain Reaction
PD-L1: programmed cell death-Ligand 1
smMIPs: single-molecule molecular inversion probes
VEGF: vascular endothelial growth factor
